# A Comparative Evaluation of Accuracy of New-generation Electronic Apex Locator with Conventional Radiography to determine Working Length in Primary Teeth: An *in vivo* Study

**DOI:** 10.5005/jp-journals-10005-1403

**Published:** 2017-02-27

**Authors:** K Vidya Bhat, Prakashchandra Shetty, Latha Anandakrishna

**Affiliations:** 1 Assistant Professor, Department of Pedodontics and Preventive Dentistry, Mathrushri Ramabai Ambedkar Dental College and Hospital, Bengaluru Karnataka, India; 2 Professor, Department of Pedodontics and Preventive Dentistry, Faculty of Dental Sciences, M.S. Ramaiah University of Applied Sciences, Bengaluru, Karnataka, India; 3 Professor and Head, Department of Pedodontics and Preventive Dentistry, Faculty of Dental Sciences, M.S. Ramaiah University of Applied Sciences, Bengaluru, Karnataka, India

**Keywords:** Conventional radiograph, Electronic apex locator, Working length.

## Abstract

**Aims:**

The purpose of this study was to evaluate the accuracy of a new-generation electronic apex locator (iPex) to determine working length in primary teeth with or without root resorption as compared with the conventional radiographic method.

**Materials and methods:**

A sample of 30 primary posterior teeth which are indicated for pulpectomy were selected for the study. After obtaining the informed consent from the parents, local anesthesia was administered. Access cavity was prepared with no.10 round bur. Initial exploration of the canals was done with no.10 K-file. Pulp was extirpated with a barbed broach followed by thorough irrigation of the canals with 0.9% saline. Initially, working length was obtained with iPex (new-generation by Nakanishi International) apex locator using no.10 K-file, which was then compared with conventional radiographic method (Ingle’s method).

**Results:**

A total of 65 canals were available for the measurement. The data were analyzed using Statistical Analysis system and t-tests were carried out. There was no statistically significant difference found when using iPex apex locator for working length determination as compared with that of conventional radiographic method (p = 0.511).

**Conclusion:**

Working length determined by iPex apex locator is comparable with that of conventional radiographic method, hence, can be used as an alternative in determining the working length of primary teeth.

**How to cite this article:**

Bhat KV, Shetty P, Anandakrishna L. A Comparative Evaluation of Accuracy of New-generation Electronic Apex Locator with Conventional Radiography to determine Working Length in Primary Teeth: An *in vivo* Study. Int J Clin Pediatr Dent 2017;10(1):34-36.

## INTRODUCTION

Biomechanical preparation, irrigation, and obturation form the key triad for a successful endodontic therapy which ultimately depends on the precise working length. In primary teeth, it is important to estimate the exact root canal length during endodontic therapy to avoid injury to the succedaneous tooth bud.^1^ Use of conventional radiography as a method of determining the working length has shortcomings, and it depends on a child’s cooperation, the operator’s proficiency, and exposure to recurrent radiation.

The electronic apex locator (EAL) is one of the breakthroughs that brought electronic science into the traditionally empirical endodontic practice. Electronic apex locators are particularly useful when the apical portion of the canal is obscured by certain anatomic structures, such as impacted teeth, tori, the zygomatic arch, excessive bone density, overlapping roots, or shallow palatal vaults. Electronic apex locators do not produce pain, help to reduce the treatment time, and help avoid unnecessary radiation which makes it more superior in pediatric endodontic procedures.^2^ Thus, they are recommended for endodontic treatment in children.

The purpose of this study was to evaluate the accuracy of a new-generation EAL (iPex) in determining working length in primary teeth with or without root resorption with that of conventional radiographic method.

## MATERIALS AND METHODS

A sample of 30 primary posterior teeth, which are indicated for pulpectomy, were selected for the study. Informed consent was obtained from each parent before the study was initiated. A standard intraoral periapical radiograph was taken using paralleling technique as a diagnostic tool to determine the periapical pathosis and the status of the root.

After local anesthetic administration, access cavities were prepared using a no.10 round bur in a high-speed handpiece. Decuspation of the teeth were done to obtain a flat occlusal surface, for standardization of the reference point. After the initial exploration of the canals with no.10 K-file, pulp was extirpated with a barbed broach followed by thorough irrigation of the canals with 0.9% saline. Finally, the access cavity was thoroughly dried with cotton pellets before using EAL.

iPex was used according to the manufacturer’s instructions. The clip was applied to the patient’s lip and no.10 K-file (Densply India Pvt. Ltd., India) connected to the electrode of the device was apically advanced in the canal, until it reached the previously calibrated 0.5 mm sign on the screen of the device, which is accepted as the apical constriction. At the meter’s 0.5 reading, the length of the file was measured and the value recorded.

Conventional radiographic measurements were made according to Ingle’s method by the operator. The file was introduced into the canal till the tentative working length which was obtained from the preoperative radiograph. Pediatric film (Kodak, E-speed film) was used in the study with an exposure of 0.6 second. Paralleling radiographic technique was followed.

No.10 K-file of 21-mm length was used, so as to avoid possible loss of apical structures due to repetitive passage of instruments. Electronic measurements were taken by a different operator so as to avoid operator bias.

### Statistical Analysis

The working length readings recorded were tabulated, and the values were subjected to statistical analysis. The data were analyzed using Statistical Analysis system and t-tests were carried out. Statistical significance was considered to be p ≤ 0.05.

## RESULTS

A total of 65 canals were available for the measurement. According to t-test, the mean values for radiographic method were 12.56 ± 1.93 mm and for EAL were 12.34 ± 1.86 mm (Table 1). There was no statistically significant difference found when using EAL as compared with that of conventional radiographic method (p = 0.51) for working length determination in primary teeth. Table 2 explains the frequency and percentage distribution. Around 20% (n = 13) of the measurements have shown no difference, in the range ±0 to 0.5 mm n = 33 (50.8%), in the range ±0.5 to 1 mm n = 13 (20%), and the measurements greater than ±1 mm were n = 6 (9.2%).

**Table 1:** **Table 1:** Means and SDs of the measurements

*Groups*		*n*		*Mean +SD*		*p-value*	
Radiographic		65		12.56 ± 1.92		0.51	
Electronic apex locator		65		12.34 ± 1.86			

n: Number; SD: Standard deviation

**Table 2:** **Table 2:** Frequency distribution with percentage of values

*Variation from radiographic apex*		*Frequency*		*Percentage*	
>1 mm		3		4.6	
0.51 to 1 mm		2		3.1	
0 to 0.5 mm		10		15.4	
0.0		13		20	
0 to -0.5 mm		23		35.4	
–0.51 to –1 mm		11		16.9	
<–1 mm		3		4.6	
Total		65		100	

Positive values indicate readings beyond the radiographic apex. Negative values indicate readings short of the radiographic apex. Value 0.0 indicates radiographic apex. Frequency indicates the number of cases out of the total sample with working lengths within a particular range from the radiographic apex.

## DISCUSSION

Locating the appropriate apical position always has been a challenge in clinical endodontics. The cement-dentinal junction, where the pulp tissue changes into the apical tissue, is the most ideal physiologic apical limit of the working length.^2^ Working length determination step in pediatric endodontics is very crucial because of possible damage to their successors due to over instrumentation and overfilling.^3-6^

The only accepted, available, and reliable method of working length determination is conventional radiography. But it has short comings, such as image distortion, superimposition of roots and/or anatomical structures like the presence of underlying permanent tooth buds, exposure to ionizing radiation, increased appointment time, and patient management. The other important problem associated with intraoral periapical radiograph is the positioning of the film inside the mouth, processing the film, and its storage.^6-8^

Electronic apex locators are the alternatives to radiographs in working length determination during endodontic therapy. Though they have been used since 1962, the first and second generations of EALs were unable to give accurate readings in the presence of irrigants, excessive hemorrhage, pus, and pulpal tissue, whereas the newer generations of EAL give reliable results in the presence of intracanal irrigants and tissues. They are also painless, easy and fast to operate, give good accurate results, and are able to detect artificial perforations.^7,9^

In the present study, accuracy of iPex was 70.8% within ±0.5 mm and 90.8% within ±1 mm. Our study results are in accordance to the study conducted by Dan-dempally et al^10^ and Nelson-Filho et al.^3^ In case of primary teeth, the apical end point of root canals is often uncertain because of continuous root resorption and remodeling. When using the apex locators, ±0.5 mm is considered acceptable by Angwaravong and Panitvisai,^11^ whereas ±1 mm is considered acceptable by others.^4,5^ The results of various studies on accuracy of different apex locators in primary teeth vary in the range of 70 to 95.82%.^6,8,10,12^

There are a number of factors that may affect electrical measurement of the root canal length: (1) The apical foramen is often located laterally, rather than apicocentral, because of the presence of physiologic root resorption in case of primary teeth; (2) it is difficult to identify the position of the apical foramen on the radiograph when it is located short of the radiographic apex on the facial or lingual aspect of the root; and (3) there is a probability of getting an electronic working length reading which is longer than radiographic reading, when the root canal curvature is in buccolingual direction. Radiographic assessment of small areas of resorption is difficult particularly in cases where resorption occurs on the buccal or lingual aspects of the root. This will often not be visible radiographically, resulting in an increased risk of overinstrumentation and/or overfilling.^13^ In such cases of resorption, working length readings with EALs would be lower than radiographic readings.

A study on the accuracy of EAL in primary teeth with root resorption showed that although the apical foramen was resorbed and enlarged, the conical shape of the canal was still maintained.^11^ The EAL (Root ZX) was capable of functioning accurately in primary teeth with resorption because the root canal typically had a decreasing taper toward the defect.^14^ In the event of discrepancies between the measurement approaches, preferences should go to the electronically determined value, provided there was a small difference (less than 2 mm) between radiographic and electronic working length readings. In cases where there is a large difference between the readings of more than 2 mm, it may be essential to take an additional working length radiograph to rule out any root canal perforations or device malfunctions.^15^ To avoid short circuit of the measuring device, it is important to do the procedure under rubber dam isolation, and the access cavity should be dried with cotton pellet before measuring.

## CONCLUSION

Working length determined by iPex apex locator is comparable with that of conventional radiographic method, hence, can be used as an alternative in determining the working length of primary teeth. The use of EAL will be useful for protecting children from exposure to recurrent ionizing radiation, over instrumentation, overfilling, damage to the permanent tooth germs, discomfort associated with film placement, and in cases where radiographic determination of root lengths has some limitations.
